# Outcomes of Treatment for Melanoma Brain Metastases

**DOI:** 10.1155/2020/7520924

**Published:** 2020-11-05

**Authors:** Mantas Janavicius, Nadezda Lachej, Giedre Anglickiene, Ieva Vincerzevskiene, Birute Brasiuniene

**Affiliations:** ^1^Department of Radiation Oncology, National Cancer Institute, Vilnius, Lithuania; ^2^Department of Medical Oncology, National Cancer Institute, Vilnius, Lithuania; ^3^Medical Statistics and Analysis Department, National Cancer Institute, Vilnius, Lithuania; ^4^Vilnius University, Faculty of Medicine, Vilnius, Lithuania

## Abstract

**Background:**

Historically, melanoma with brain metastases has a poor prognosis. In this retrospective medical record review, we report basic clinicopathological parameters and the outcomes of patients with melanoma and brain metastases treated with different treatment modalities before the era of immunotherapy and modern radiotherapy technique.

**Methods:**

Patients with metastatic melanoma were treated with surgery, radiotherapy, and/or systemic therapy from 1998 to 2017. In our study, they were identified and stratified depending on treatment methods. Overall survival was defined as the time from the date of brain metastases to the death or last follow-up (2019 June 1^st^). Survival curves were estimated using the Kaplan–Meier method that was employed to calculate the hazard ratio.

**Results:**

Six (12%) of 50 patients are still alive as of the last follow-up. The median overall survival from the onset of brain metastases was 11 months. The longest survival time was observed in patients treated by surgery followed by radiotherapy, surgery followed by radiotherapy and systemic therapy, and also radiotherapy followed by systemic therapy. The shortest survival was observed in the best supportive care group and patients treated by systemic therapy only.

**Conclusions:**

Patients with brain metastases achieved better overall survival when treated by combined treatment modalities: surgery followed by radiotherapy (26.6 months overall survival), combining surgery, radiotherapy, and systemic therapy (18.7 months overall survival), and also radiotherapy followed by systemic therapy (13.8 months overall survival).

## 1. Background

### 1.1. Epidemiology

Melanoma is a malignant tumor of melanocytes, which are cells that make the pigment melanin and are derived from the neural crest. Melanoma brain metastases (MBM) are an increasingly common clinical challenge. Lung, breast cancers and melanoma are the most common cancers leading to metastases in the brain. However, melanoma has the highest propensity.

Brain metastases develop in nearly half of patients with advanced melanoma, representing a cause of death in up to 54%. [1]. Five-year cumulative incidence of brain metastases was around 7% for patients with all stages of melanoma [2]. Approximately 20% of all melanoma patients have brain metastases at first diagnosis of distant metastatic melanoma [3]. Additionally, up to 45% of patients with metastatic melanoma develop clinically documented brain metastases during their lifetime [4], and the prevalence of brain metastasis is 50–75% in the autopsy series [5, 6].

Melanoma patients with brain metastases historically have had a poor prognosis with a median survival of 4 months and a 1-year survival rate of 10–20% [7, 8].

Improvement in median overall survival from 8 to 10 months has been reported with stereotactic radiosurgery (SRS) [9, 10].

In melanoma patients with brain metastases, a good performance status and a limited number of brain metastases were associated with a more favourable prognosis [11].

Melanoma 5-year survival varies from 97% (stage IA disease) to 40% (stage IIIC). Metastatic melanoma 5-year survival is about 15% [12]. In a study presented by Vosoughi, the median time from primary melanoma diagnosis to brain metastasis was 3.2 years and the median overall survival duration from the time of initial brain metastasis was 12.8 months [13].

### 1.2. Treatment Options for the Melanoma Patients with Brain Metastases

Traditional systemic therapy with such chemotherapy agents like dacarbazine, paclitaxel, or carboplatin has limited activity in melanoma brain metastases because of complicated drug delivery to the brain due to the blood-brain barrier (BBB). Some alkylating agents, such as temozolomide, lomustine, and fotemustine, which are known to have good BBB penetration, have been investigated in patients with melanoma brain metastases, but the results showed modest efficacy [14–20].

The discovery of BRAF V600 mutation and the development of targeted therapies directed against this mutation as well as effective immunotherapies with durable benefits have revolutionized the treatment of patients with melanoma.

Mutations of BRAF, NRAS, and KIT are three common mutations seen in metastatic melanoma [21, 22]. Approximately 50% of melanomas harbor a mutation in the BRAF, mostly confined to a specific point mutation at nucleotide 1799, leading to a change in the V600 amino acid [23].

The presence of BRAF or NRAS mutations increases the risk of central nervous system (CNS) metastases in patients with advanced melanoma [24]. Dabrafenib and vemurafenib target the BRAF V600 mutation. These agents and their combination with MEK inhibitors are approved for the treatment of metastatic melanoma.

There were several prospective and retrospective clinical trials, where the efficacy of targeted therapy with or without radiotherapy was evaluated in melanoma patients with brain metastases. The results showed median intracranial progression-free survival (PFS) from 2 to 6 months approximately, median overall survival (OS), 4–12 months [25–31]. Clinical trials in this area are ongoing in order to find out the feasibility, safety, and effectiveness of combining BRAF inhibitors and radiation therapy.

Since melanoma is known to be an immunogenic malignancy, novel immunotherapy agents have been developed recently. The effectiveness of immune checkpoint inhibitors (cytotoxic T-lymphocyte-associated antigen 4 (CTLA-4) and programmed death 1 (PD-1)) and targeted therapies (BRAF–MEK inhibitors) in melanoma patients with brain metastases have made a significant change in melanoma treatment. These agents have intracranial activity in patients with melanoma who have untreated brain metastases and may improve survival outcomes[6, 7]. Ipilimumab, which blocks cytotoxic T-lymphocyte antigen 4, and pembrolizumab or nivolumab, antiprogrammed death 1 agents, have been shown to be active against brain metastases from melanoma when each agent is used individually as monotherapy. [32–34].

Randomized phase 2 clinical study CheckMate 204 evaluated the efficacy of the nivolumab and ipilimumab treatment combination in patients with melanoma who have asymptomatic, untreated brain metastases. In this study, 94 patients were randomized. The median follow-up was 14.0 months; the rate of intracranial clinical benefit was 57% (95% confidence interval [CI], 47 to 68); the rate of complete response was 26%; the rate of partial response was 30%; the rate of stable disease for at least 6 months was 2%. So this combination of medications really gives more hope for melanoma patients with brain metastases. [35].

More recent trials of systemic treatments in patients with melanoma have shown CNS responses similar to extracerebral responses. [36–38].

Surgery is mainly limited to patients with solitary or single brain metastasis and is often performed for symptomatic relief. Compared with radiation therapy alone, an overall survival benefit in all patients with single brain metastasis who undergo resection has been demonstrated: median survival was 9.2 months for patients who received surgery as compared with 3.5 months for patients who received radiotherapy alone [39]. There may also be a role for resection in the oligometastatic disease of dominant, symptomatic lesions. As the field comes closer to achieving integrated histologic and genetic diagnoses for these patients, a secondary benefit of debulking is the procurement of adequate tumor tissue for molecular characterization. Investigators in one study compared the genomics of matched brain metastases and primary tumors across multiple histologies and demonstrated that more than 50% of brain metastases harbored genetic alterations that were not detected in the clinically sampled primary tumor [40].

Standard whole-brain radiation therapy treatment regimens have established a total dose of 30 Gy fractionated over 2 to 3 weeks. In randomized studies, whole-brain radiation therapy after resection or radiosurgery did not confer an overall survival benefit; however, this therapy did improve intracranial disease control and lessened the risk of death secondary to neurologic causes [41].

Dissimilar to whole-brain radiation therapy, the efficacy of SRS is not influenced by the primary tumor of origin. As with surgical resection, stereotactic radiosurgery has been most beneficial in achieving local control of small (<3 cm) lesions in patients with fewer than three total lesions [42]. Other factors that impact the decision to pursue surgery versus stereotactic radiosurgery include lesion location. Neoadjuvant radiosurgery is one of the treatment options for patients with a limited number of brain metastases. However, neoadjuvant SRS shows similar outcomes as adjuvant SRS including overall survival, local control, and distant control. The major potential advantage for neoadjuvant SRS appears to be decreasing the risk of leptomeningeal disease and symptomatic radiation necrosis. [42].

Recent preclinical and clinical data suggest that radiotherapy may be a promising combination partner for immunomodulatory agents and, in particular, for immune checkpoint inhibitors [43]. Additionally, ipilimumab treatment was associated with significantly prolonged overall survival compared to patients receiving cranial radiotherapy and other systemic treatment [44]. The optimal sequence of radiotherapy and immunotherapy is currently still not clear, although there is limited evidence that concurrent treatment may be beneficial for OS and intracranial disease control compared to radiotherapy after ipilimumab. However, combining SRS and immunotherapy can increase the incidence rate of symptomatic radiation-induced brain necrosis [45].

## 2. The Aim

The aim of our study is retrospectively to compare and evaluate the effectiveness of specific therapies, including surgery, radiotherapy, and systemic therapy, as well as combinations of these treatment methods for the treatment of the melanoma brain metastases.

This study was approved by the Institutional Review Board.

## 3. Methods

The authors analyzed the medical data of melanoma patients with brain metastases between the year 1998 and the year 2017 treated at the National Cancer Institute (Vilnius), one of the largest specialized oncology centers in Lithuania. Melanoma is a relatively rare malignancy, but the incidence is increasing every year. In the year 2012, 313 new melanoma cases were registered in Lithuania, and 43 of these cases were in the advanced stage. Melanoma ranks 15th in the number of new cases among men and women and remains a major cause of mortality. A total number of 50 patients were identified as eligible and included in this analysis. The survival status of all patients was confirmed. We last updated the patients' data in June 2019. The clinicopathological characteristics of the patients are shown in Table 1.

23 men (46%) and 27 women (54%) were included in our study. Six of all patients (12%) were still alive on the date of follow-up.

A group of patients (*n* = 15) were treated with surgery due to brain metastases. It was performed mainly for patients with single brain metastases and often for symptomatic relief.

The majority of the patients (*n* = 35) were treated with radiotherapy. For most patients, whole-brain radiotherapy was performed and only two patients were candidates for stereotactic radiotherapy. The standard whole-brain radiotherapy regimen was 30 Gy/10 fractions. Stereotactic radiotherapy was performed by a single 18 Gy fraction.

Some patients (*n* = 27) were treated with adjuvant (interferon alfa-2b, *n* = 13, 48,1%) and/or palliative systemic therapy (52%, chemotherapy or targeted therapy) before the brain metastases were diagnosed. Among the chemotherapy agents were dacarbazine (*n* = 15, 55.6%), a combination of carboplatin and paclitaxel (*n* = 3, 11.1%), CVD (Cisplatin, Vinblastine, Dacarbazine) regimen (*n* = 1, 3.7%), and targeted therapy including vemurafenib ± cobimetinib or dabrafenib ± trametinib (*n* = 6, 22.2%). After brain metastases were diagnosed, 16 patients received systemic treatment alone or with other treatment methods (Table 2). The regimen depended on whether the patient was treated or not with systemic therapy before. The principal chemotherapy agents were dacarbazine, temozolomide, or carboplatin and paclitaxel. Other patients were treated by targeted therapy with vemurafenib ± cobimetinib or dabrafenib ± trametinib and just one patient was treated with interferon alfa-2b. Most of the patients, after brain metastases were diagnosed, received one line of the chemotherapy and only one patient received two lines of treatment (carboplatin with paclitaxel, then temozolomide). Immunotherapy was available in our country only from the year 2015 but not reimbursed at that time, so it was not administered.

Patients were stratified in these categories: brain surgery (performed or no surgery), radiotherapy (performed or no radiotherapy), systemic therapy (performed or no systemic therapy), and solitary or multiple CNS metastases. Different treatment modalities after diagnosis of melanoma are presented in Table 3.

We also analyzed the overall survival variability from different treatment modalities. The outcomes considered in this study were overall survival, 1-year survival, and 2-year survival.

### 3.1. Statistical Analysis

The survival rate estimates with the 95% CI were calculated using the Kaplan–Meier method.

## 4. Results

The median patient's age on the day of diagnosis in all patients was 54 years (21–81 years). 43 patients (86%) had a diagnosis of cutaneous melanoma, 6 (12%) melanoma of unknown primary, and 1 patient (2%) intraocular melanoma. BRAF mutations were confirmed for 8 patients (16%). For 35 patients (70%), BRAF status was not identified, and the remaining 7 patients (14%) did not have BRAF mutations. Mutations were detected from a formal fixed paraffin-embedded surgical material or biopsy. DNA was first purified and then the quantified PCR method was used. Information about BRAF status is shown in Table 4.

**Figure 1:**
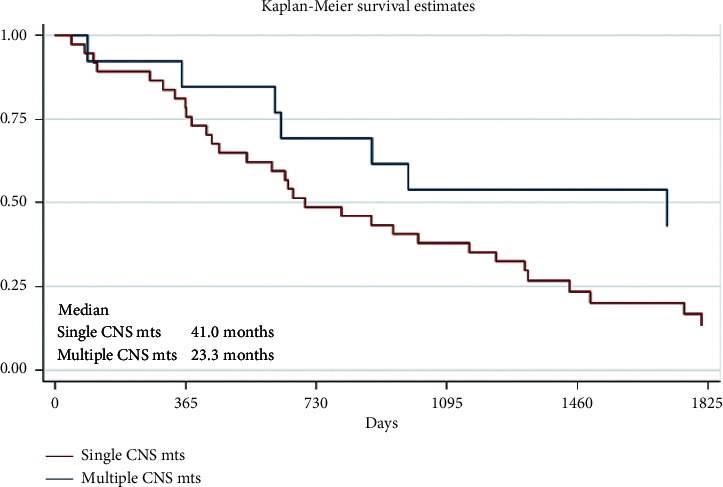
Survival results depending on the number of brain metastases (*p*=0.02).

Most of the patients (37 patients, 74%) had multiple metastases in the brain, and just 13 patients (26%) had solitary brain metastases. The survival results of the groups are presented in Table 5 and Figure 1.

**Figure 2:**
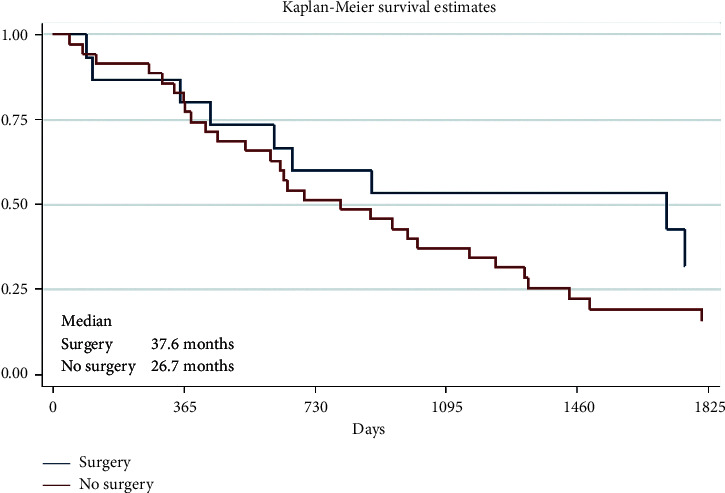
Survival results of the patients treated by surgery (*p*=0.13).

Fifteen (30%) of 50 patients had surgery due to brain metastases. The survival results of these two groups are presented in Table 6 and Figure 2.

**Figure 3:**
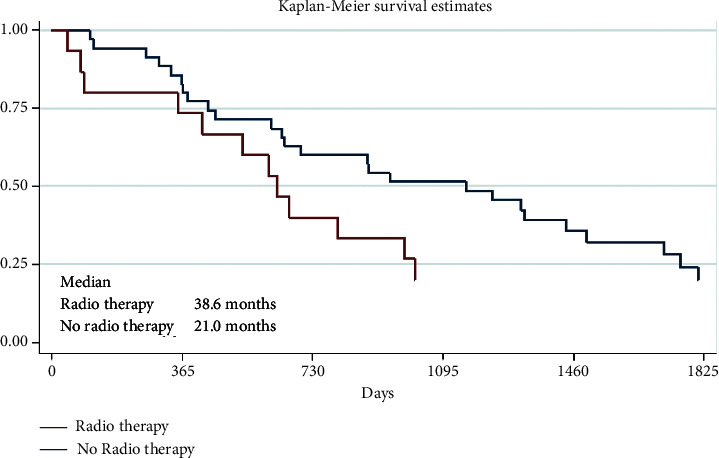
Survival results of the patients treated by radiotherapy (*p*=0.34).

More than half (*n* = 35, 70%) of all 50 patients received radiotherapy. The survival results of these two groups are presented in Table 7 and Figure 3.

**Figure 4:**
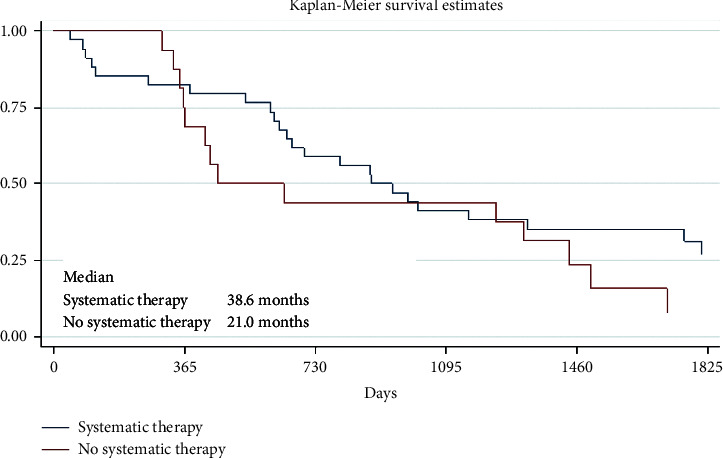
Survival results of the patient treated by systemic therapy (*p*=0.46).

Sixteen (32%) of 50 patients received systemic therapy. The survival results of these groups are presented in Table 8 and Figure 4.

**Figure 5:**
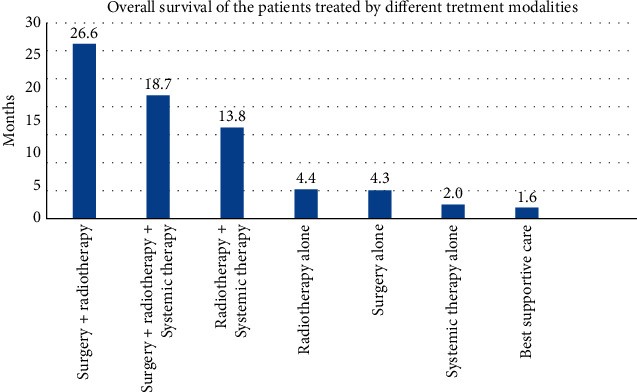
Overall survival of the patients treated by different treatment modalities.

Median survival from the onset of the brain metastases in patients with melanoma was 11 months. The longest overall survival was observed in these treatment modalities: surgery followed by radiotherapy (26.6 months), surgery followed by radiotherapy and systemic therapy (18.7 months), and radiotherapy followed by systemic therapy (13.8 months). The shortest survival was observed in the best supportive care group and patients treated by systemic therapy alone. Survival results by different treatment modalities are presented in Figure 5.

## 5. Discussion

Our analysis focused on the subgroup of patients with melanoma CNS metastases in order to evaluate survival results from different treatment modalities. The female gender in our study was slightly more frequent (54% vs. 46%) than the male gender, which is different from the gender distribution in other reports. The median age at the time of diagnosis was 54 years, which is similar compared to other studies.

Historically, the prognosis of patients with melanoma brain metastases is poor, with a median OS of 4–6 months. Better OS is expected in the era of modern systemic therapies and local therapy with SRS. The Melanoma Institute in Australia included 355 patients diagnosed with melanoma brain metastases from January 2011 to December 2014. The median OS was 7.1 months (95% confidence interval [CI] 6.0–8.1). Median OS differed by treatment modality: systemic therapy and SRS and/or surgery 14.9 months (95% CI 10.7–19.0), SRS and/or surgery with or without whole-brain radiotherapy (WBRT) 6.4 months (95% CI 5.4–7.5), systemic therapy 5.4 months (95% CI 3.1–7.7), systemic therapy and WBRT 5.2 months (95% CI 4.1–6.4), WBRT 4.4 months (95% CI 2.4–6.3), and best supportive care 1.8 months (95% CI 1.2–2.3). Similar tendencies were observed in our analysis in the National Cancer Institute. OS for patients with melanoma brain metastases appears improved in the modern therapy era, particularly for patients who are candidates for systemic therapy with SRS or surgery.

Sandru [45] presented a retrospective study in the Journal of Medicine and Life in the year 2014. They collected data of all patients with metastatic melanoma treated in the Budapest Oncologic Institute between 2008 and 2013. In one of the subgroups, there were 27 patients with brain metastases. The longest OS was 9 months (40.7 OS was 3 months, 18.5%–6 months and 9.9%–9 months). A discussion about using treatment methods was not presented in this publication, but if to compare with our data, the median overall survival in our clinic was longer during the same period.

The median survival of patients in our study is similar to other studies. Also, based on other studies, V600E mutation is the most common BRAF mutation in melanoma, occurring in 70–90% of BRAF mutant melanomas. We also found a similar number of these mutations in our study, 75% of *BRAF* mutant melanomas carried the V600E mutation.

In our analyses, overall survival using radiotherapy alone was slightly better than in the best supportive care group. The true impact of WBRT on brain metastases from melanoma is likely to be limited. Although the efficacy of WBRT is controversial, its toxicity is well documented in the form of neurocognitive decline manifested by memory loss and impaired executive function. The rapid uptake of SRS revolutionized the care of patients with brain metastases. In the case of melanoma, the lethal dose of radiation delivered with SRS appears sufficient to kill melanoma cells. SRS can effectively lead to local control of established brain metastases, but its use is limited by the number of presented metastases. Although many centers are treating multiple lesions now, the accepted standard and published literature support the use of SRS for up to only three lesions.

Analyzing the 1-, 2-, or 5-year survival results, in our study, there were no big differences between 1-year OS results if there were solitary or multiple CNS metastases, whether there was the surgical treatment of CNS metastases or not and radiotherapy or systemic therapy was administered or not. However, much more interesting and important, what differences were seen after 5 years when longer OS was observed in patients with the solitary CNS metastases group (OS 43.1% vs. 13.4% for multiple CNS metastases) and for those who received systemic therapy (OS 27.3% vs. 7.8% without systemic therapy) or had a brain metastases surgery (OS 32.0% vs. 15.9% without surgery), but there was no difference after 5 years between the patients' group who had WBRT or without WBRT (OS 20.1% vs. 20.0%, respectively). It seems that this treatment method is not significant for the OS of patients with melanoma CNS metastases. Nowadays, WBRT has very narrow indications for melanoma patients with CNS metastases. Therefore, SRS is preferred instead of WBRT as the initial therapy for patients with melanoma brain metastases.

In our analysis, we had 13 patients (26%) with solitary CNS metastases. However, the time in which patients were treated and observed was a factor for that when WBRT was applied to most patients. In 2018, the National Cancer Institute (Vilnius, Lithuania) started to use a new linear accelerator with the opportunity to perform stereotactic radiotherapy.

We should mention that immunotherapy has been reimbursed for melanoma patients in Lithuania just since July 2018, just as modern radiotherapy was introduced in 2018. We expect that immunotherapy and SRS will change the future results of our melanoma patients with brain metastases.

Immunotherapy has transformed the treatment of metastatic melanoma, with 3-year survival rates surpassing 50% for eligible patients [46]. However, many of these patients still require surgery, but very little research has been done on this group of patients to determine whether surgery after immunotherapy conveys any benefits. Danielle M. Bello, MD, of Memorial Sloan Kettering Cancer Center, and colleagues designed a study to examine surgical intervention outcomes for these patients. Its findings were presented at the 2018 Society of Surgical Oncology (SSO) Annual Cancer Symposium. The median overall survival for the entire group was 23 months, while overall survival for the group that received immunotherapy and surgery was 21 months.

Brain metastases are a common clinical occurrence in patients with metastatic melanoma. The prognosis for patients with brain metastases from metastatic melanoma remains poor. The ongoing development of systemic therapy makes the improvement in CNS control even more important. The number of metastases in the brain is prognostically relevant. Regarding the fact that the treated lesion is responsible for the progress as well as new lesions, the radiotherapeutic approach should initially be stereotactic radiotherapy or whole-brain radiotherapy in some cases. Modern technology makes it possible to potentially reduce late side effects.

In a vast majority of metastatic melanoma cases, deaths observed are due to disease progression in the brain. Surgery is the most effective method for patients with solitary CNS metastases. In most cases in our study, patients received postoperative radiotherapy, and the longest overall survival was observed in this group (surgery + radiotherapy). The main factors that made systemic therapy less effective were because there were hardly any innovative drugs, and most of the all patients treated with systemic therapy got chemotherapy (only one-third of all patients treated by systemic therapies received targeted therapy, no immunotherapy was given). We expect a longer survival of patients with brain metastasis in the era of targeted therapy and immunotherapy.

All new modern treatment methods, such as SRS, targeted therapies, and immunotherapy, are improving the survival results of metastatic melanoma. At present, you can find about 100 active clinical studies that are searching for new optimal treatment methods for melanoma patients with brain metastases. One of the main study objects now is how to combine immunotherapy and SRS. These studies found that concurrent SRS and immunotherapy shows meaningful intracranial activity in patients with either asymptomatic or symptomatic melanoma brain metastases [47].

Other active studies are trying to find out how to combine targeted therapies and immunotherapy and how to use the newest medications or SRS in a neoadjuvant setting.

## 6. Conclusions

This retrospective analysis suggests that our analyzed patients with brain metastases achieve a better overall survival when treated by combined treatment modalities: surgery followed by radiotherapy (26.6 months overall survival), combining surgery, radiotherapy, and systemic therapy (18.7 months overall survival), and also radiotherapy followed by systemic therapy (13.8 months overall survival).

However, it is important that an individualized medical strategy be developed to manage melanoma brain metastases, with a multidisciplinary team of radiation oncologists, neurosurgeons, medical oncologists, neurologists, and radiologists, and palliative care specialists.

## Figures and Tables

**Table 1:** Clinicopathological characteristics of the patients.

Characteristics	Number of patients

Gender	
Women	27 (54%)
Men	23 (46%)

Age	
<30	2 (4%)
30–50	19 (38%)
51–70	21 (42%)
>71	8 (16%)

BRAF mutation	
Unknown	35 (70%)
(+)	8 (16%)
(-)	7 (14%)

Number of brain metastases	
1	13 (26%)
>1	37 (74%)

Surgery (brain metastases)	
(+)	15 (30%)
(-)	35 (70%)

Radiotherapy	
(+)	35 (70%)
(-)	15 (30%)

Primary melanoma localisation:	

-Cutaneous	43 (86%)
-Uveal	1 (2%)
-Unknown primary	6 (12%)

Systemic therapy (after a diagnosis of brain metastases)	
(+)	16 (32%)
(-)	34 (68%)

**Table 2:** Systemic treatment after diagnosis of brain metastases.

Type of systemic treatment (1^st^ line)	Number of patients	Duration of treatment
Chemotherapy		
Dacarbazine	5 (31.2%)	2–6 cycles
Temozolomide	3 (18.7%)	1–3 cycles
Carboplatin with paclitaxel	1 (6.2%)	6 cycles
Targeted therapy with vemurafenib ± cobimetinib or dabrafenib ± trametinib	6 (37.5%)	6–16 months
Interferon alfa-2b	1 (6.2%)	6 months

**Table 3:** Treatment modalities after diagnosis of melanoma.

Treatment method	Number of patients
Surgery + radiotherapy	9 (18%)
Radiotherapy alone	13 (26%)
Systemic therapy + radiotherapy	10 (20%)
Surgery alone	3 (6%)
Systemic therapy alone	2 (4%)
Surgery + radiotherapy + systemic therapy	6 (12%)
Best supportive care	7 (14%)

**Table 4:** BRAF mutations in melanoma patients.

BRAF mutations	Number of patients
V600K	1 (12.5%)
V600M	1 (12.5%)
V600E	6 (75%)

**Table 5:** Survival results depending on the number of the metastases.

Group	Number of patients	1-year survival	2-year survival	5-year survival
Solitary CNS metastases	13 (26%)	85.6% [51.8; 96.2]	69.2% [37.3; 87.2]	43.1% [15.6; 68.3]
Multiple CNS metastases	37 (74%)	78.4% [61.4; 88.6]	48.7% [32.0; 63.4]	13.4% [4.5; 27.2]

**Table 6:** Survival results of the patients treated by surgery.

Group	Number of patients	1-year survival	2-year survival	5-year survival
Surgery	15 (30%)	80.0% [50.0; 93.1]	60.0% [31.8; 79.7]	32.0% [8.9; 58.5]
No surgery	35 (70%)	80.0% [62.6; 89.9]	51.4% [34.0; 66.4]	15.9% [6.0; 30.1]

**Table 7:** Survival results of the patients treated by radiotherapy.

Group	Number of patients	1-year survival	2-year survival	5-year survival
Radiotherapy	35 (70%)	82.9% [65.8; 91.9]	60.0% [42.0; 74.0]	20.1% [7.9; 36.2]
No radiotherapy	15 (30%)	73.3% [43.6; 89.1]	40.0% [16.5; 62.8]	20.0% [4.9; 42.4]

**Table 8:** Survival results of the patients treated by systemic therapy.

Group	Number of patients	1-year survival	2-year survival	5-year survival
Systemic therapy	16 (32%)	82.3% [64.9; 91.7]	58.8% [40.6; 73.2]	27.3% [13.2; 43.5]
No systemic therapy	34 (68%)	75.0% [46.3; 89.8]	43.8% [19.8; 65.6]	7.8% [0.0; 29.1]

## Data Availability

All the data are collected from the National Cancer Institute of Lithuania.

## Abbreviations

MBM: Melanoma brain metastases
SRS: Stereotactic radiosurgery
BBB: Blood-brain barrier
CNS: Central Nervous System
PFS: Progression-free survival
OS: Overall survival
CTLA-4: Cytotoxic T-lymphocyte-associated antigen 4
PD-1: Programmed death 1
CI: Confidence interval
Gy: Gray
DNA: Deoxyribonucleic acid
PCR: Polymerase chain reaction
WBRT: Whole-brain radiotherapy
SSO: Society of Surgical Oncology.
